# The Safety of a Conservative Fluid Replacement Strategy in Adults Hospitalised with Malaria

**DOI:** 10.1371/journal.pone.0143062

**Published:** 2015-11-18

**Authors:** Ne Myo Aung, Myat Kaung, Tint Tint Kyi, Myat Phone Kyaw, Myo Min, Zaw Win Htet, Nicholas M. Anstey, Mar Mar Kyi, Josh Hanson

**Affiliations:** 1 Insein General Hospital, Yangon, Myanmar; 2 Hpa-an Hospital, Hpa-an, Kayin State, Myanmar; 3 Department of Medical Research (Lower Myanmar), Yangon, Myanmar; 4 Myanmar Medical Association, Yangon, Myanmar; 5 Menzies School of Health Research, Charles Darwin University, Darwin, Australia; Erasmus Medical Centre, NETHERLANDS

## Abstract

**Background:**

A conservative approach to fluid resuscitation improves survival in children with severe malaria; however, this strategy has not been formally evaluated in adults with the disease.

**Methods:**

Adults hospitalised with malaria at two tertiary referral hospitals in Myanmar received intravenous fluid replacement with isotonic saline, administered at a maintenance rate using a simple weight-based algorithm. Clinical and biochemical indices were followed sequentially.

**Results:**

Of 61 adults enrolled, 34 (56%) had *Plasmodium falciparum* mono-infection, 17 (28%) *Plasmodium vivax* mono-infection and 10 (16%) mixed infection; 27 (44%) patients were at high risk of death (*P*. *falciparum* infection and RCAM score ≥ 2). In the first six hours of hospitalisation patients received a mean 1.7 ml/kg/hour (range: 1.3–2.2) of intravenous fluid and were able to drink a mean of 0.8 ml/kg/hour (range: 0–3). Intravenous fluid administration and oral intake were similar for the remainder of the first 48 hours of hospitalisation. All 61 patients survived to discharge. No patient developed Adult Respiratory Distress Syndrome, a requirement for renal replacement therapy or hypotension (mean arterial pressure < 60mmHg). Plasma lactate was elevated (> 2 mmol/L) on enrolment in 26 (43%) patients but had declined by 6 hours in 25 (96%) and was declining at 24 hours in the other patient. Plasma creatinine was elevated (> 120 μmol/L) on enrolment in 17 (28%) patients, but was normal or falling in 16 (94%) at 48 hours and declining in the other patient by 72 hours. There was no clinically meaningful increase in plasma lactate or creatinine in any patient with a normal value on enrolment. Patients receiving fluid replacement with the conservative fluid replacement algorithm were more likely to survive than historical controls in the same hospitals who had received fluid replacement guided by clinical judgement in the year prior to the study (p = 0.03), despite having more severe disease (p < 0.001).

**Conclusions:**

A conservative fluid resuscitation strategy appears safe in adults hospitalised with malaria.

## Background

Fluid therapy is integral to the care of all critically ill patients, but despite decades of spirited debate, the optimal strategy for the fluid management of adults with severe malaria remains incompletely defined [1–3]. Hypovolaemia is almost universally present in these patients on admission to hospital [4, 5] and it has seemed intuitive to correct this fluid deficit promptly. Early restoration of the circulating volume might be expected to help remedy the lactic acidosis and acute kidney injury (AKI) which are common in this population and strong predictors of death [6]. However, the primary pathogenesis of the lactic acidosis and AKI seen in falciparum malaria is at the microvascularlevel, including obstruction by parasitised red blood cells (pRBCs) [7–10], a process relatively unaffected by fluid loading [7, 9]. Of even more significance to clinicians is the fact that adults with severe malaria also have increased capillary permeability and can develop the acute respiratory distress syndrome (ARDS), even while hypovolaemic [9, 11]; malaria related ARDS is frequently fatal even if mechanical ventilation is available [12].

The World Health Organization (WHO) guidelines for the management of severe malaria between 1990 and 2010 recognised the clinical challenge of fluid resuscitation in this population, but in the absence of any evidence to guide them, provided no specific advice for clinicians [13–15]. However the most recent 2014 WHO guidelines cautiously proposed that patients who are not shocked, do not appear severely dehydrated, and are not anuric on admission, should receive 3–5 ml/kg/hour of normal saline during the first 6 hours of hospitalisation (cumulative total 18–30 ml/kg) with patients checked for basal crepitations or an increase in work of breathing every 2 hours [16]. These more detailed recommendations are a welcome guide for clinicians, however they still represent a relatively generous fluid load. In the landmark FEAST study, which examined the fluid management of children with severe febrile illness and impaired perfusion, the patients randomised to the maintenance fluid arm received a cumulative median fluid load of only 10.1 ml/kg in the first eight hours and had a lower mortality than those receiving intravenous boluses of saline and albumin [17]. While it is not possible to simply extrapolate these paediatric data to adults, they raise the concern that in an adult with an adequate blood pressure and urine output–as is usually the case in severe malaria [18]—the potential for harm from the fluid load recommended in the most recent WHO guidelines may outweigh any putative benefits.

This observational study of adults hospitalised with malaria–which commenced before publication of the 2014 WHO guidelines–assessed the response of adults to a conservative weight-based fluid resuscitation strategy. It was anticipated that more cautious fluid loading would result in few cases of ARDS, but given the potential for this strategy to exacerbate renal function and acid-base status, biochemical indices were closely followed to confirm the safety of such an approach.

## Methods

### Patients

The study was performed between April 1 2014 and September 30, 2015 at two tertiary referral hospitals in Lower Myanmar: Insein Hospital in Yangon and Hpa-an Hospital in Kayin State. The two hospitals serve populations in which there is low perennial transmission of malaria, with a peak in transmission during the wet season which runs from April to September.

Initially it was planned that only patients hospitalised with *P*. *falciparum* infection would be enrolled in the study, however due the higher than expected incidence of *P*. *vivax* hospitalisations, the limited data on the response of these patients to fluid resuscitation and the risk of ARDS in vivax malaria [19], it was decided in June 2014 that patients requiring hospitalisation for infection with any species of malaria should be eligible for enrolment.

**Fig 1:**
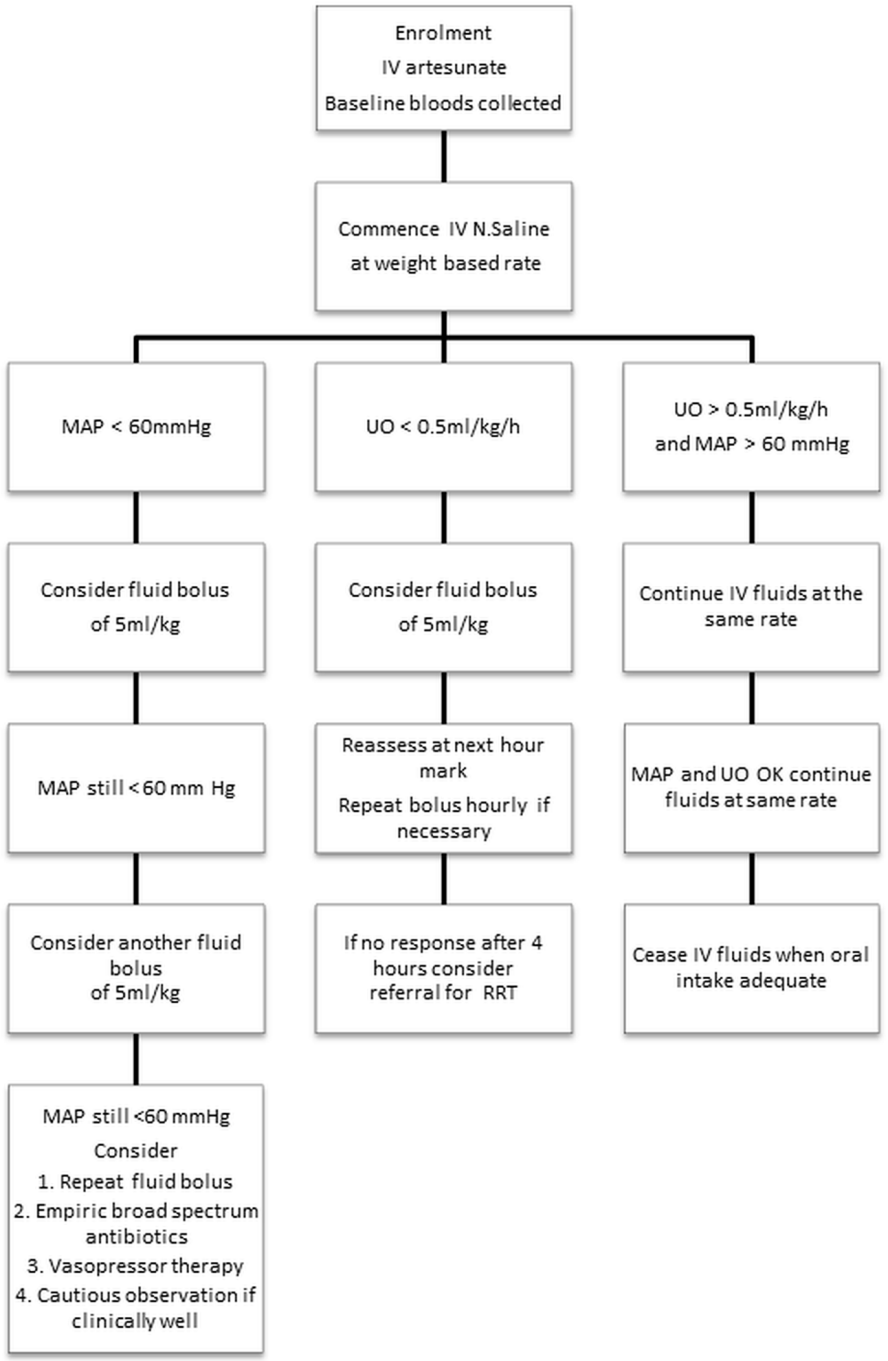
Fluid management algorithm used in the study. Ideal body weight determined using patient height (see Table 1) IV: Intravenous, MAP: Mean arterial pressure, UO: Urine output, RRT: Renal replacement therapy.

Patients with suggestive clinical symptoms were tested with immunochromatographic tests (SD Bioline Malaria Ag P.f/P.v (05FK80), Standard Diagnostics, Republic of Korea). The diagnosis was later confirmed by the finding of asexual forms on a simultaneously collected blood film. Patients were excluded from the study if they were <16 years of age, pregnant, had an adequate oral intake or had received >48 hours of parenteral anti-malarial therapy. Patients were also excluded if they were haemodynamically shocked (systolic blood pressure <80mmHg with cool peripheries) or anuric on admission. Patients with ARDS (oxygen saturation <90% on room air with bibasal crepitations) on admission were also excluded as diuresis—rather than intravenous fluid therapy—is recommended in these patients. Patients with a haemoglobin of <7 g/dL on admission were excluded if they could receive a blood transfusion in the next 6 hours (as blood rather than intravenous crystalloid is the recommended intravenous fluid in these patients). Study participants received intravenous artesunate and, apart from the conservative fluid resuscitation algorithm (Fig 1), standard supportive care was provided as per current national Myanmar treatment guidelines for malaria [20] which recommend intravenous artesunate in all patients and adjunctive antibacterial therapy at the discretion of the treating clinician.

**Table 1:** Formula used to calculate weight-based fluid infusion rate [21].

Ideal body weight (kg)*	4	6	8	10	12	14	16	20	30	40	50	60	70
Infusion rate (ml/hour)	16	24	32	40	44	48	52	60	70	80	90	100	100

* determined using the formula: Females: 45.5 kg + 0.9 kg/cm for each cm > 152 cm; Males: 50 kg + 0.9 kg/cm for each cm > 152 cm).Weight rounded up—or down—to the nearest 10kg; maximum infusion rate 100ml/hour.

### Ethics

The study received ethical approval from the Ethics Committee of the Department of Medical Research (Lower Myanmar), Ministry of Health, Government of the Republic of Myanmar and the Human Research Ethics Committee of the Menzies School of Health Research in Darwin, Australia. As patients were acutely unwell on enrolment, the ethics committees agreed that they lacked the capacity to provide informed consent. Written informed consent was therefore instead sought from an adult (18 years of age or older) family member. An adult family member provided written informed consent for the eight patients aged 16 to 18 years who were enrolled in the study.

### Patient management

Patients were cared for by local doctors working on the medical wards of both hospitals. All cases had a full medical history taken and a physical examination performed. Clinical indices of hypovolaemia (dry mucous membranes, reduced tissue turgor, reduced jugular venous pressure, tachycardia and history of a reduced urine output) were specifically sought and were used by the admitting clinicians to categorise patients as euvolaemic or as mildly, moderately or severely hypovolaemic. This classification however had no influence on the fluid that was administered to patients who instead received normal (0.9%) saline using a relatively simple algorithm based on ideal body weight (Fig 1, Table 1). Maintenance fluid was prescribed using published weight-based formulae [21], but for ease of application the patients’ weight was rounded up or down to the nearest 10 kg. As only adults were enrolled in the study, this meant for practical purposes that all patients received intravenous fluid at a rate between 80 and 100 ml/hour.

The patients’ clinical condition and fluid balance was assessed hourly for the first six hours, then six-hourly until 48 hours. After this time patients received usual ward care. Venous blood was collected on admission, at six hours, 24 hours and 48 hours and at the clinicians’ discretion thereafter. Biochemical and haematological indices were measured using point of care devices (iStat, Abbott) or automated analysers in the hospitals. Disease severity was determined using the RCAM score [6].

Prior to the study’s commencement, there had been no protocol for fluid management of malaria at either hospital; instead medical staff used their clinical judgement to determine the rate of fluid delivery. Therefore to further assess the safety of the conservative fluid strategy, the study’s case-fatality rate was compared with the case-fatality rate in a clinical audit of patients admitted with malaria at the two hospitals in the year before the study began [22].

### Statistics

Statistical analysis was performed using statistical software (Stata 10.0, Statacorp). Groups were analysed using the Kruskal-Wallis and Chi-squared tests. For the purposes of the analysis, patients with mixed *P*. *falciparum*/*P*. *vivax* infection were classified as having *P*.*falciparum* infection.

## Results

**Fig 2:**
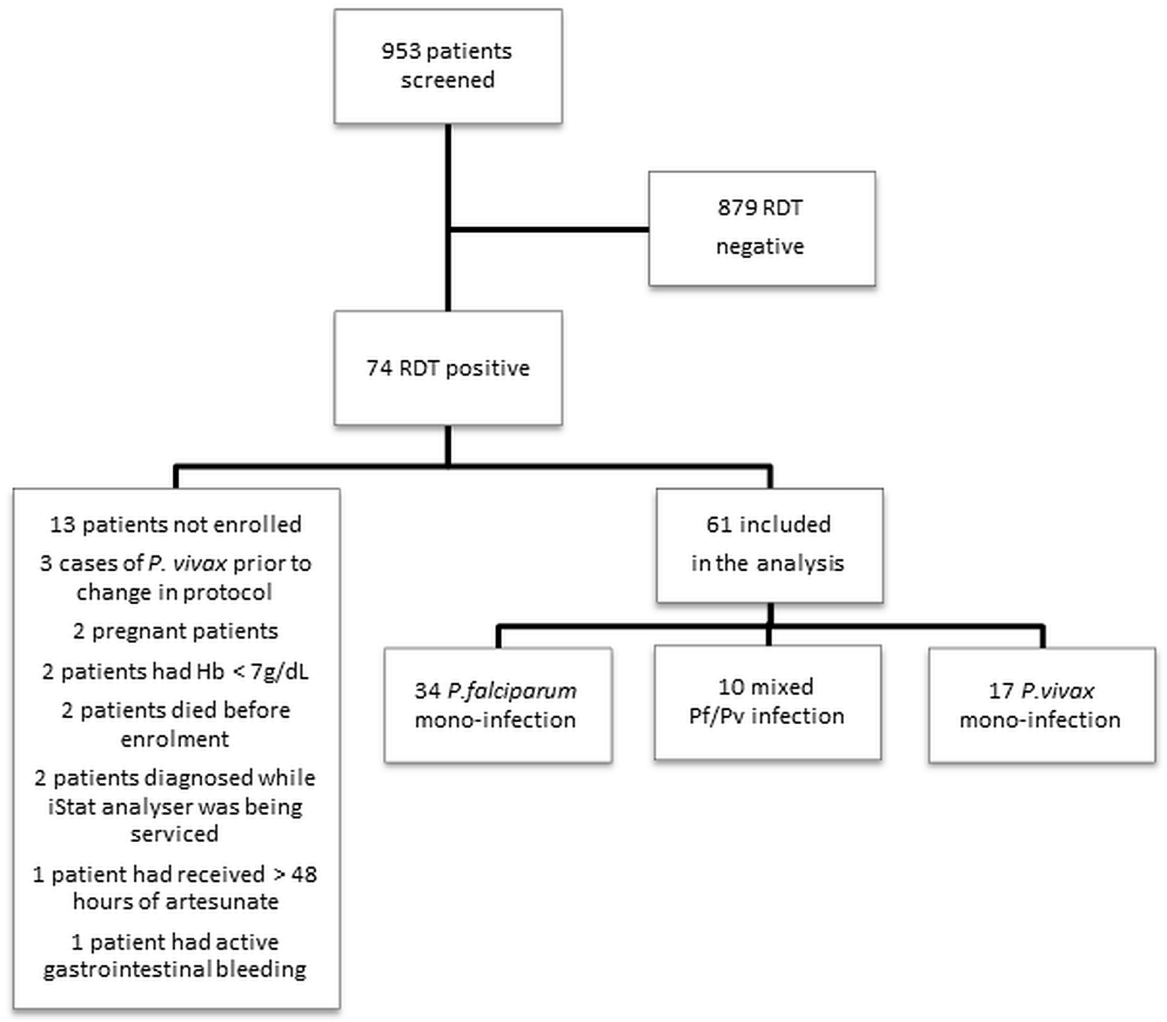
Screening and enrolment. RDT: Rapid diagnostic test. Hb: Haemoglobin, Pf: *P*.*falciparum*, Pv: *P*.*vivax*.

61 patients were enrolled at the two sites (Fig 2), 39 in Yangon and 22 in Hpa-an. The characteristics of the patients were similar at both sites. Of the 61 patients, 34 (56%) had *P*. *falciparum* mono-infection, 17 (28%) had *P*. *vivax* mono-infection and 10 (16%) had mixed *P*. *falciparum/P*. *vivax* infection. On admission, 30 (49%) patients could be classified as at high-risk of a complicated course (RCAM score ≥ 2), 27 (90%) of whom had *P*. *falciparum* infection (p = 0.002). Of the 17 patients hospitalised with *P*. *vivax* mono-infection, 14 (82%) had an RCAM score of 1. The remaining three (18%) had an RCAM score of 2, all of whom were tachypnoeic (respiratory rates of 32, 36 and 44 breaths per minute), one of these three patients had unilateral consolidation on chest x-ray suggesting superadded bacterial infection. None of the three was hypoxic (oxygen saturations of 96%, 98% and 98% on room air). None of these three patients had coma (GCS < 11), but two had impaired consciousness (a GCS of 12 and 13 respectively).

All 61 patients survived to discharge. The mean duration of hospitalisation was 5.3 days (95% confidence interval (95% CI): 4.8–5.8); patients infected with *P*. *falciparum* had a mean hospital stay of 5.7 days (95% CI: 5.0–6.3) versus 4.3 days (95% CI: 3.6–5.0) in patients with *P*. *vivax* mono-infection (p = 0.02). On enrolment 10 (16%) patients were classified as clinically euvolaemic, 37 (61%) as mildly hypovolaemic, 13 (21%) as moderately hypovolaemic, while 1 patient was felt to be severely hypovolaemic. All patients were treated with intravenous artesunate and 48 (79%) were treated with intravenous antibiotics (most of whom—40 (83%)—received a third generation cephalosporin).

### Volume of fluid received and fluid balance

Using the weight-based algorithm, patients received a mean 1.7 ml/kg/hour (95% CI 1.7–1.7 range: 1.3–2.2) of intravenous fluid in the first 6 hours of hospitalisation. During this time patients were also able to consume a mean of 0.8 ml/kg/hour (95% CI 0.6–1.0, range: 0–3); the mean total fluid intake over the first 6 hours was therefore 2.5 ml/kg/hour (95% CI 2.3–2.7, range: 1.5–4.8). The rate of intravenous fluid administration and oral ingestion were similar over the ensuing 42 hours (Table 2). Although the fluid algorithm permitted the administration of fluid boluses for hypotension and oliguria, the hypotension trigger was never reached. Hourly urine output was difficult to quantify in some cases as almost all conscious patients declined a urinary catheter but only two patients had a urine output below 0.5 ml/kg/hour after the first 6 hours and in only one of these did this last for more than 24 hours. This patient had a urine output of 20 ml in the first 6 hours, 200 ml in the first 24 hours and 400 ml in the first 48 hours, but she was clinically euvolaemic and improving and so was managed without fluid boluses; she was discharged well with normal renal function after a 5 day hospitalisation. The mean cumulative fluid balance in the 61 patients of the study was +445 ml (95% CI: 365–526) in the first 6 hours, +1525 ml (95% CI: 1326–1724) in the first 24 hours and +2612 ml (95% CI: 2285–2940) in the first 48 hours.

**Table 2:** Intravenous fluid administration and oral intake over the first 48 hours of the study.

***P*. *falciparum* infection (n = 44)**						
	Intravenous		Oral		Total	
	ml	ml/kg/h	ml	ml/kg/h	ml	ml/kg/h
**First 6 h**	571 (558–584)	1.7 (1.7–1.8)	240 (169–310)	0.8 (0.5–1.0)	811 (746–876)	2.5 (2.2–2.7)
**First 24 h (cumulative)**	2287 (2237–2337)	1.7 (1.7–1.8)	964 (793–1134)	0.7 (0.6–0.9)	3251 (3084–3418)	2.5 (2.3–2.6)
**First 48 h (cumulative)**	4278 (4058–4498)	1.6 (1.5–1.7)	2230 (1919–2540)	0.9 (0.7–1.0)	6508 (6158–6858)	2.5 (2.3–2.6)
***P*. *vivax* mono-infection (n = 17)**						
	Intravenous		Oral		Total	
	ml	ml/kg/h	ml	ml/kg/h	ml	ml/kg/h
**First 6 h**	585 (568–603)	1.7 (1.7–1.8)	332 (241–424)	1.0 (0.7–1.2)	918 (819–1018)	2.7 (2.4–2.9)
**First 24 h (cumulative)**	2308 (2228–2389)	1.7 (1.6–1.8)	1250 (971–1529)	0.9 (0.7–1.1)	3558 (3239–3878)	2.6 (2.4–2.8)
**First 48 h (cumulative)**	4249 (3823–4674)	1.6 (1.4–1.7)	2515 (2048–2983)	0.9 (0.8–1.1)	6764 (6276–7253)	2.5 (2.3–2.7)
**All patients (n = 61)**						
	Intravenous		Oral		Total	
	ml	ml/kg/h	ml	ml/kg/h	ml	ml/kg/h
**First 6 h**	575 (565–586)	1.7 (1.7–1.7)	266 (209–322)	0.8 (0.6–1.0)	845 (790–900)	2.5 (2.4–2.7)
**First 24 h (cumulative)**	2293 (2251–2334)	1.7 (1.7–1.7)	1040 (895–1185)	0.8 (0.7–0.9)	3318 (3170–3466)	2.5 (2.4–2.6)
**First 48 h (cumulative)**	4270 (4080–4461)	1.6 (1.5–1.7)	2306 (2050–2561)	0.9 (0.8–1.0)	6576 (6294–6858)	2.5 (2.3–2.6)

All numbers represent mean (95%CI)

### Renal function and plasma electrolytes

**Fig 3:**
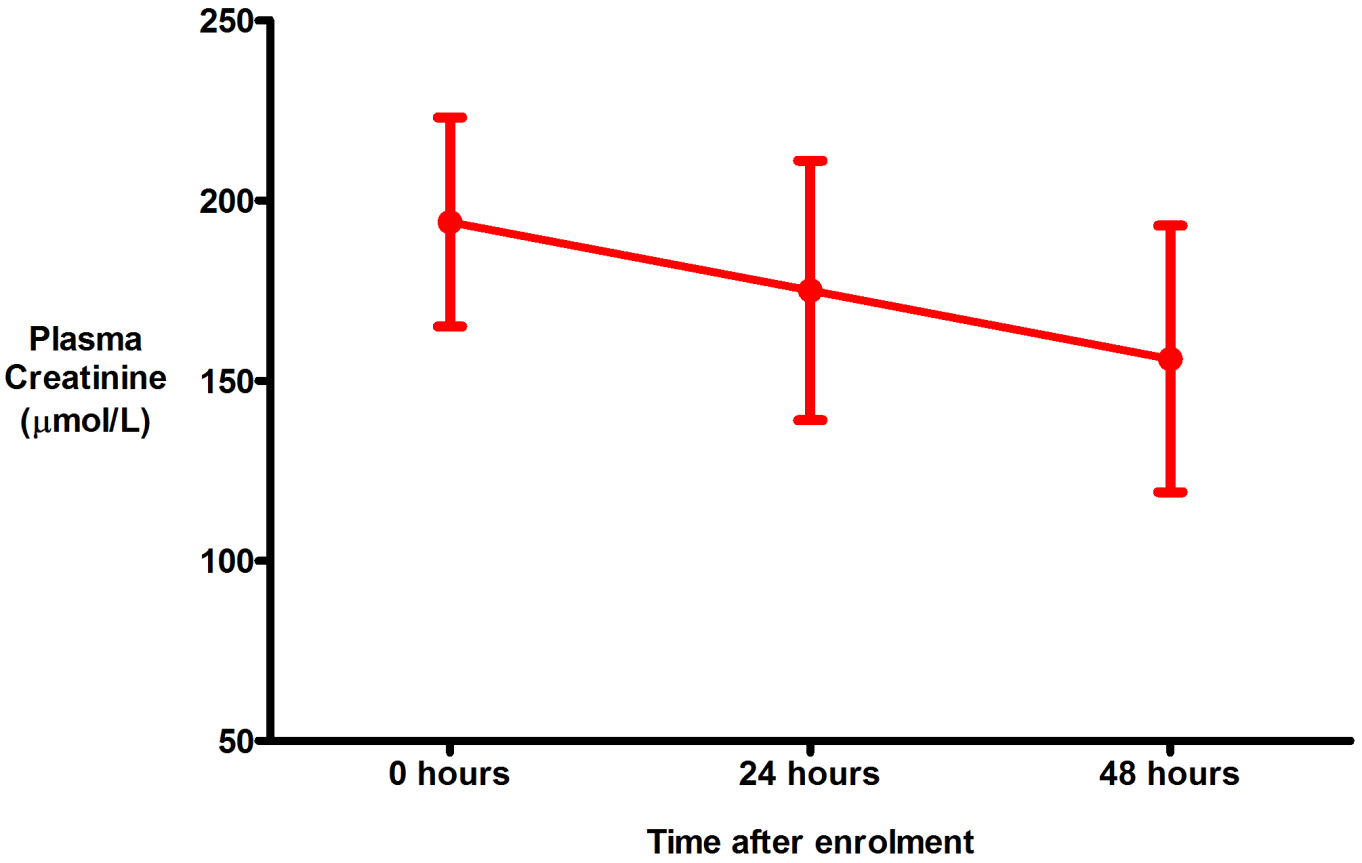
Response in plasma creatinine to the conservative fluid strategy (includes only patients with an elevated plasma creatinine (>120 μmol/L) on enrolment). The data points represent the mean and the error bars the standard error of the mean.

An elevated plasma creatinine (>120 μmol/L) was present on enrolment in 17 (28%) patients, all of whom had *P*. *falciparum* infection (p = 0.003). Plasma creatinine fell over the course of the study in all these patients (Fig 3). It fell within the first 24 hours in 12 (71%), within 48 hours in another 4 (24%) and within 72 hours in the remaining patient. Three patients with *P*. *falciparum* infection and a normal creatinine on admission developed an abnormal creatinine after 24 hours; in two it had normalised again by 48 hours. In the third patient the creatinine rose from 86 μmol/L on admission to 201 μmol/L at 24 hours, before falling again to 135 μmol/L at 48 hours; as this patient was clinically improving and had a urine output over 1 ml/kg/hour over this entire period, it was felt safe to observe him without intervention despite the rise in creatinine. No patient required renal replacement therapy (RRT) during the study. There was no clinically significant change in electrolyte concentrations during the study, particularly plasma chloride (Table 3).

**Table 3:** Change in clinical and laboratory variables over the first 48 hours of the study.

***P*. *falciparum* infection (n = 44)**			
	Enrolment	24 hours	48 hours
Glasgow Coma Score	14 (13–14)	15 (14–15)	15 (15–15)
Temperature (°C)	38.8 (38.5–39.2)	37.6 (37.4–37.9)	37.2 (37.0–37.3)
Mean arterial pressure (mmHg)	79 (76–83)	83 (80–85)	83 (80–86)
Pulse (beats/minute)	98 (93–104)	89 (84–93)	83 (80–85)
Oxygen saturation (%)	97 (97–97)	98 (97–98)	98 (97–98)
Respiratory rate (breaths/minute)	33 (31–35)	25 (24–27)	21 (19–22)
Plasma sodium (mmol/L)	137 (135–139)	137 (136–139)	139 (137–141)
Plasma potassium (mmol/L)	3.7 (3.5–3.8)	3.5 (3.3–3.6)	3.6 (3.5–3.7)
Plasma chloride (mmol/L)	102 (100–105)	103 (102–105)	104 (102–106)
Blood urea (mmol/L)	8.5 (6.6–10.4)	6.5 (4.8–8.2)	6.0 (4.1–7.9)
Plasma creatinine (μmol/L)	125 (97–154)	117 (85–150)	105 (75–136)
Plasma glucose (mmol/L)	6.4 (6.0–6.9)	6.7 (6.1–7.2)	6.1 (5.8–6.4)
Venous pH	7.39 (7.36–7.42)	7.41 (7.40–7.42)	7.39 (7.38–7.41)
Plasma bicarbonate (mmol/L)	21.9 (20.7–23.1)	22.5 (21.5–23.4)	23.9 (22.8–24.9)
Plasma lactate (mmol/L)	2.7 (2.0–3.4)	1.3 (1.1–1.5)	1.1 (1.0–1.3)
Base deficit (mmol/L)	3 (2–4)	2 (1–3)	1 (0–2)
Haemoglobin (g/dL)	11.1 (10.3–12.0)	10.1 (9.4–10.8)	10 (9.3–10.7)
White cell count (x 10^9^/L)	6.3 (5.6–7.0)	-	-
Platelet count (x 10^9^/L)	98 (74–121)	-	-
***P*. *vivax* mono-infection (n = 17)**			
	Enrolment	24 hours	48 hours
Glasgow Coma Score	15 (14–15)	15 (15–15)	15 (15–15)
Temperature (°C)	39.2 (38.7–39.6)	37.3 (36.9–37.6)	37.1 (36.8–37.4)
Mean arterial pressure (mmHg)	87 (81–93)	83 (78–87)	84 (80–87)
Pulse (beats/minute)	102 (91–113)	87 (81–93)	83 (78–89)
Oxygen saturation (%)	98 (97–98)	98 (98–99)	98 (97–98)
Respiratory rate (breaths/minute)	28 (25–31)	23 (20–26)	20 (18–22)
Plasma sodium (mmol/L)	136 (133–139)	139 (136–142)	139 (137–141)
Plasma potassium (mmol/L)	3.8 (3.5–4.1)	3.5 (3.3–3.7)	3.8 (3.5–4.1)
Plasma chloride (mmol/L)	102 (100–104)	103 (100–105)	103 (101–105)
Blood urea (mmol/L)	4.7 (3.9–5.5)	3.8 (2.4–5.1)	4.2 (2.8–5.5)
Plasma creatinine (μmol/L)	93 (85–100)	78 (67–89)	78 (72–84)
Plasma glucose (mmol/L)	6.7 (5.9–7.6)	6.5 (5.9–7.1)	6.6 (6.0–7.2)
Venous pH	7.40 (7.37–7.42)	7.40 (7.37–7.42)	7.40 (7.38–7.41)
Plasma bicarbonate (mmol/L)	24.1 (22.5–25.7)	25.4 (23.8–27.0)	25.8 (24.8–26.7)
Plasma lactate (mmol/L)	1.6 (1.3–1.9)	1.1 (0.8–1.4)	1.0 (0.8–1.2)
Base deficit (mmol/L)	1 (0–2)	0 (-2-2)	0 (-2-1)
Haemoglobin (g/dL)	11.2 (9.8–12.6)	11.5 (10.2–12.7)	10.8 (9.4–12.1)
White cell count (x 10^9^/L)	7.3 (5.9–8.8)	-	-
Platelet count (x 10^9^/L)	108 (55–161)	-	-

All numbers represent mean (95%CI)

### Lactic acidosis

**Fig 4:**
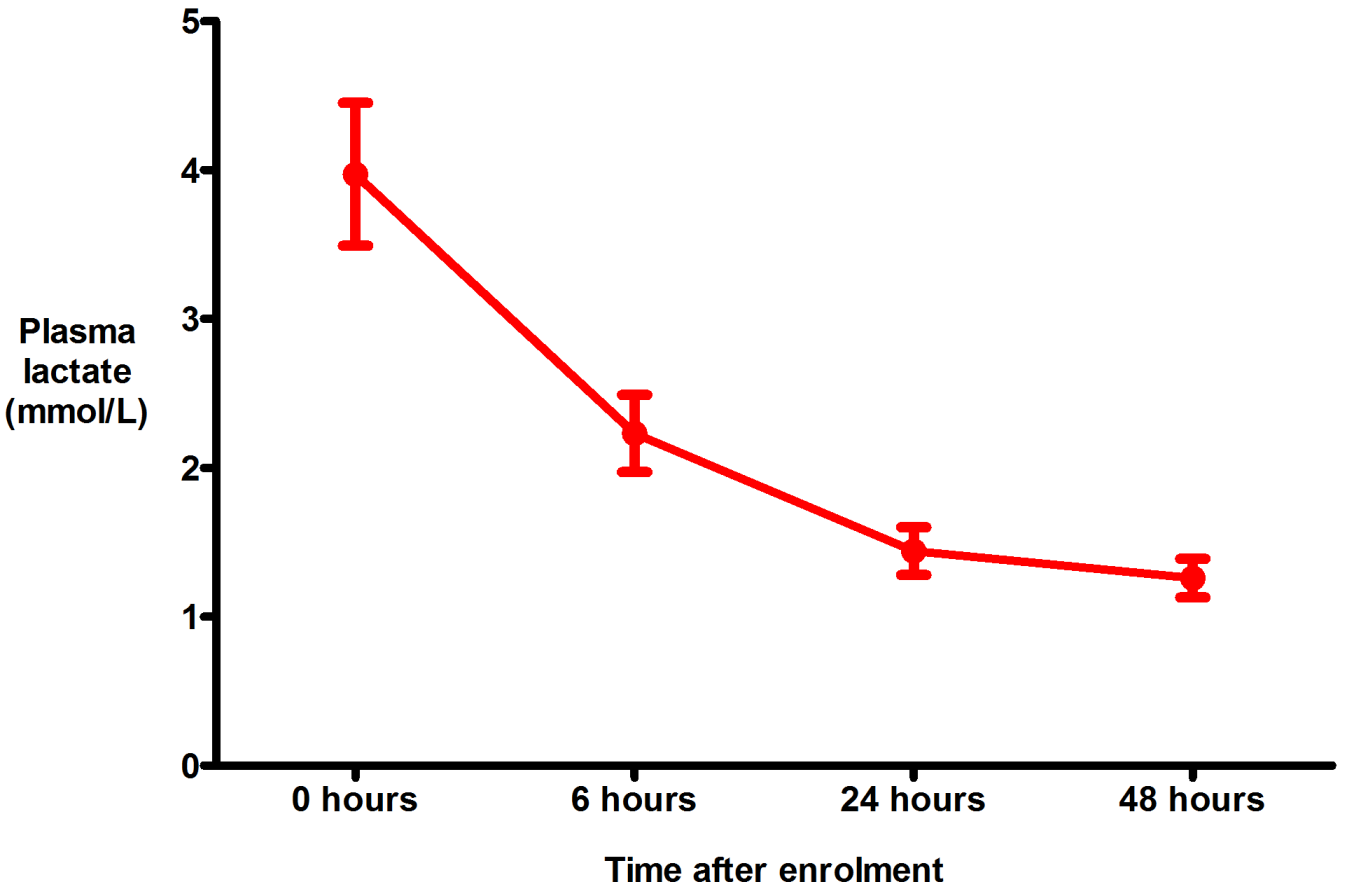
Response in plasma lactate to the conservative fluid strategy (includes only patients with an elevated plasma lactate (>2 mmol/L) on enrolment). The data points represent the mean and the error bars the standard error of the mean.

An elevated plasma lactate (>2 mmol/L), was present in 26 (43%) patients: 20 (77%) had *P*.*falciparum* infection and six had *P*.*vivax* mono-infection (p = 0.47). However the highest plasma lactate recorded in a patient with *P*.*vivax* mono-infection was 2.6 mmol/L compared with the maximum value of 13.6 mmol/L in a patient infected with *P*.*falciparum*. Plasma lactate fell promptly in almost all patients with an elevated plasma lactate on admission (Fig 4): 25 (96%) had a fall in the first 6 hours and 23 had a normal plasma lactate by 24 hours. One patient’s plasma lactate had risen at 6 hours (from 5.1 mmol/L to 5.6 mmol/L) but fell sequentially thereafter. Four (11%) patients with *P*.*falciparum* and a normal plasma lactate on enrolment developed an elevated lactate during the study (peak 3.1 mmol/L), but in three this had returned to normal by 48 hours. The fourth patient developed an elevated lactate of 2.3 mmol/L at 48 hours (compared with 1.4 mmol/L at 24 hours). The lactate was not repeated after this point, but the patient went on to make a complete recovery and was discharged well 4 days later.

### Other clinical findings

No patient developed a mean arterial pressure of less than 60 mmHg during the course of the study and no patient required vasopressor therapy. No patient developed ARDS; 4 required supplementary oxygen, but none required invasive or non-invasive ventilation.

### Comparison with previous fluid replacement strategy

**Fig 5:**
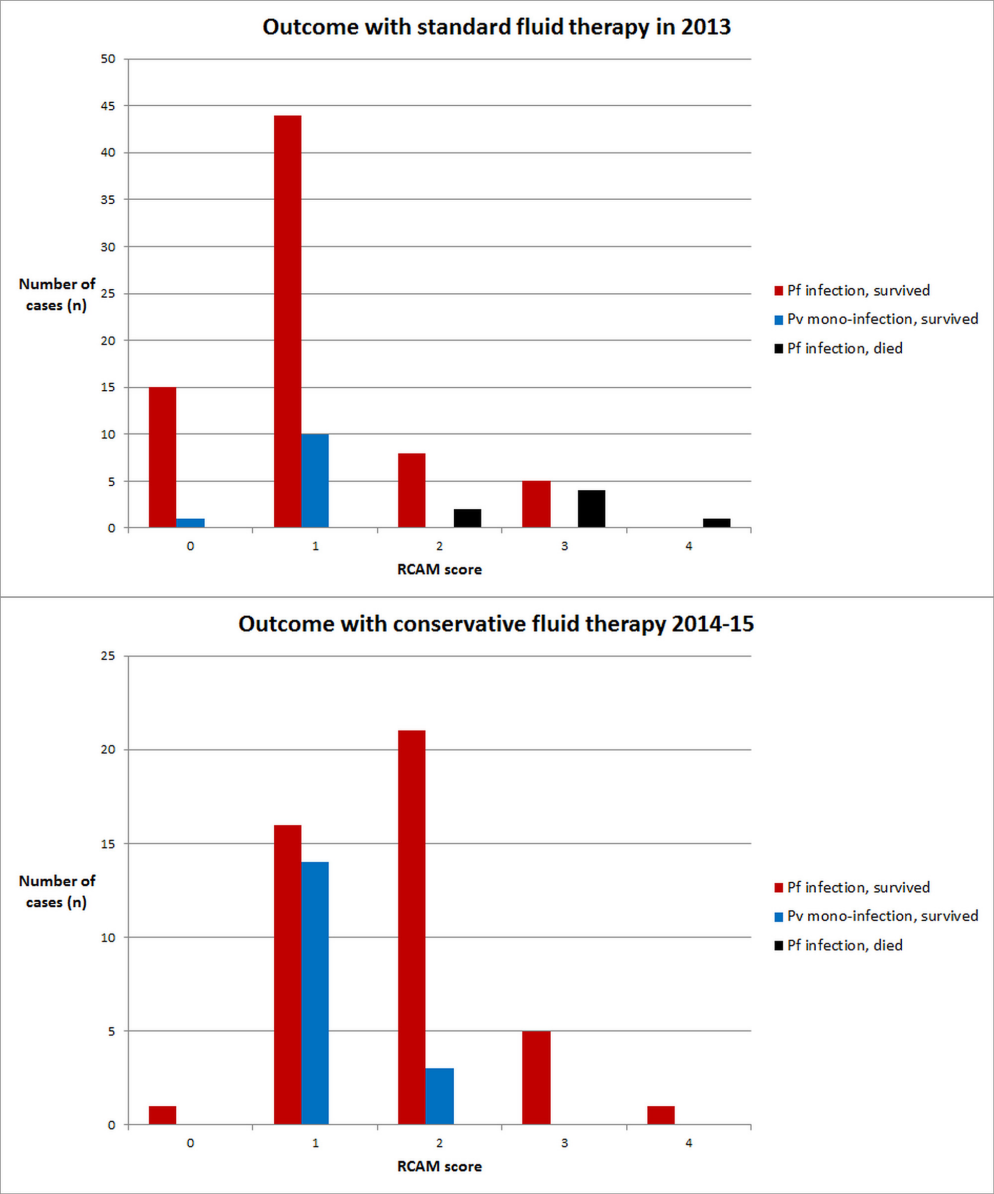
Survival rate of patients receiving conservative fluid therapy in 2014–15 compared with controls receiving standard fluid therapy at the same hospitals in 2013. (Results stratified by malaria species and RCAM score). No patient died in 2014–5; no patient with P.vivax mono-infection died in either time period.

In 2013, 90 patients were admitted to the two hospitals with a diagnosis of malaria, 7 (8%) of these patients died (Fig 5). Patients managed using the conservative fluid replacement algorithm in 2014–15 were significantly more likely to survive than this historical control group (p = 0.03), despite the fact that more patients in 2014–15 were at a high risk of death (*P*. *falciparum* infection and RCAM ≥ 2) than in 2013: 27/61 (44%) versus 19/90 (21%) (p < 0.001).

## Discussion

Although most patients were clinically hypovolaemic on admission, every patient in the study survived despite relatively conservative fluid replacement. No patient developed shock, ARDS, a requirement for RRT or a clinically significant deterioration in their acid-base status or electrolyte profile. Survival was significantly higher than that of patients who had received fluid guided by clinical judgement in the year prior to the study, despite the fact that patients in the current study had more severe disease. These findings suggest that a conservative fluid strategy is safe in adults hospitalised with malaria in the absence of another indication for fluid loading. Given that a liberal fluid strategy has been linked to harm in adults [9] and increased mortality in children [17], these data support the hypothesis that conservative fluid administration is the preferred approach in patients hospitalised with malaria.

The fundamental aim of fluid resuscitation is to improve blood flow to vital organs and accordingly it is central to the management of hypotension, oliguria and reduced tissue perfusion in a variety of critically ill populations [23–26]. While adults hospitalised with malaria are almost universally hypovolaemic [4, 5], most adults with malaria are neither hypotensive nor oliguric [18] and nearly all have a preserved cardiac output [9, 27, 28]. While the increased lactate:pyruvate ratios seen in patients with severe falciparum malaria suggest tissue hypoperfusion [29], this primarily results from mechanical obstruction of the microcirculation with pRBCs and microvascular dysfunction rather than from hypovolaemia [4]. Even with liberal fluid loading (and appropriate anti-malarial therapy) mechanical obstruction and dysfunction can persist for greater than 72 hours, continuing to impede the microvascular blood flow that the fluid loading aims to improve [7, 9, 30].

The most recent WHO guidelines propose a cumulative 18–30 ml/kg of intravenous fluid in the first 6 hours of hospitalisation [16], however this is quantitatively similar to the median cumulative volume of 40 ml/kg that was delivered over 8 hours to the two arms of the FEAST study with the highest mortality, and much greater than the median cumulative volume of 10.1 ml/kg over 8 hours in the arm with the greatest survival [17]. Given the documented harms of fluid boluses in patients with malaria and the fact that adults are usually normotensive and passing urine, it is reasonable to ask what the relatively large fluid load proposed in the 2014 WHO guidelines is aiming to accomplish.

Of course it is possible to have too restrictive a fluid therapy strategy; evolving hypovolaemia will result inevitably in impaired blood flow to vital organs and lactic acidosis, renal dysfunction and eventually hypotension. However despite the fact that over 80% of the patients had clinical evidence of hypovolaemia on enrolment and almost all had risks for increased ongoing insensible losses (fever, diaphoresis and tachypnoea), these complications did not develop to a clinically significant degree in any patient during the course of the study.

The study’s results are in line with a growing awareness in critical care literature of the hazards of fluid excess in many critical care populations [31–34] while the safety of a restrictive resuscitation strategy has been confirmed in others [35]. Although there is still debate about whether an increasing positive fluid balance is the cause—or the result—of clinical deterioration, there is a general consensus that in the presence of adequate left ventricular function and blood flow to vital organs, there is nothing to be gained with further fluid loading, even if patient is fluid responsive [36, 37]. Indeed there is the increased risk of tissue oedema and deterioration in acid-base status and gas exchange [34].

There is also growing concern about the potential association between the use of chloride-rich fluids and the development of a range of complications in critically ill patients including AKI, acidosis and coagulopathy [38, 39]. Indeed one of the hypotheses for the increased mortality seen in the patients receiving a fluid bolus in the FEAST study was the potential for hyperchloraemia to exacerbate the acid-base status and therefore vascular haemodynamics and myocardial performance [40]. In a previous study of fluid resuscitation in adults with severe falciparum malaria, patients received a mean fluid load of 3683 ml (95% CI: 2965–4400) of intravenous normal saline over 6 hours guided by transpulmonary thermodilution [9]. There was a concomitant increase in the mean plasma chloride from 103 mmol/L (95% CI: 100–105) to 109 mmol/L (95% CI: 107–112) which contributed to a fall in the mean pH of 0.06 (95% CI: 0.02–0.1) over the same time period [9]. Despite the promise of balanced fluids in critically ill populations [41], if a smaller volume of fluid–and hence chloride—is administered to these patients, any potential for harm from hyperchloraemia may become less relevant; indeed in this study there was no significant change in plasma chloride over 48 hours. Administering fluid to patients hospitalised with malaria at a maintenance rate using widely used formulae applicable to other hospital populations, also avoids the logistic challenges of having separate and unique disease management pathways [42].

The study has a number of limitations. “Severe malaria” has been defined so frequently by so many different authors that there has been some confusion about just what severe disease really “is” [43]. Using the more pragmatic definition of disease requiring hospitalisation overcomes some of this uncertainty, although that being said this cohort was less unwell than other severe malaria series [44]. Nevertheless, 27 (44%) patients in the study could be classified as at high risk of death (*P*. *falciparum* with a RCAM score ≥ 2) and the 100% survival of these patients in this study contrasts with a case-fatality rate of 37% (7/19) in this patient group at the same hospitals in 2013. While comparisons with historical controls are not conclusive, the RCAM score has been shown to be a robust and reliable predictor of outcome in adults with malaria [6, 22, 45]; the absence of a single fatality in the study despite the significant number of patients with high RCAM scores is therefore encouraging.

While the current 2014 WHO recommendations for fluid management of adults with malaria may appear to have some shortcomings, without a randomised controlled trial, they cannot be said to be inferior to the algorithm employed in this study. Indeed, although all patients received less than 2 ml/kg/h of intravenous fluid, the majority of patients were still able to ingest some fluid orally and in the first 6 hours of their hospitalisation 25% of the patients actually had a total fluid input of ≥ 3 ml/kg/h, the lower end of the WHO recommendation. Furthermore, these data cannot be applied to the patient sub-groups who were excluded from the study–although the fact that there were no patients excluded on the basis of ARDS, anuria or non-haemorrhagic shock in a study performed at two large tertiary referral hospitals, suggests that these represent a minority of adults with malaria.

## Conclusions

Liberal fluid therapy is hazardous in adults with severe malaria, and can precipitate ARDS without significantly improving the AKI and acidosis it aims to treat. This study demonstrates that a more conservative fluid strategy, using a simple weight-based algorithm, is associated with a low incidence of ARDS without appearing to lead to a clinically significant deterioration in acid-base status, renal function, electrolyte profile or systemic haemodynamics. Whilst these data require validation in larger studies of patients with more severe disease, they suggest that in the absence of another strong indication for fluid loading, a conservative fluid strategy should be the preferred approach in adults hospitalised with malaria.
